# Differential effects of L-alanine, L-glutamine, L-arginine HCl, and glycine on feed intake and hypothalamic appetite-related responses in layer-type chicks

**DOI:** 10.3389/fphys.2026.1983120

**Published:** 2026-10-27

**Authors:** Elham Ghashghaei, Ahmed Mijiyawa, Muhammad Faisal Riaz, Minghui Wang, Kelin Li, Dan Wang, Maxwell Ansong Okai, Clarice Maa Maa Temhoul, Yendouhamtchié Nadiedjoa, Behrouz Rahmani, Uchechukwu Edna Obianwuna, Morteza Zendehdel, Hai Lin

**Affiliations:** 1 Key Laboratory of Efficient Utilization of Non-grain Feed Resources (Co-construction by Ministry and Province), Ministry of Agriculture and Rural Affairs, Shandong Provincial Key Laboratory of Animal Nutrition and Efficient Feeding, Department of Animal Science, Shandong Agricultural University, Taian, Shandong, China; 2 Regional Center of Excellence for Avian Science, University of Lome, Lome, Togo; 3 Department of Internal Medicine, School of Medicine, Hazrat- e Rasool General Hospital, Iran University of Medical Sciences (IUMS), Tehran, Iran; 4 Institute of Feed Research, Chinese Academy of Agricultural Science, Beijing, China; 5 Department of Basic Sciences, Faculty of Veterinary Medicine, University of Tehran, Tehran, Iran

**Keywords:** amino acids, feed intake, hypothalamus, ICV injection, layer-type chicks

## Abstract

**Introduction:**

While the effects of L-alanine, L-glutamine, L-arginine monohydrochloride (L-arginine HCl) and glycine on feed intake (FI) have been studied in chicks, their hypothalamic neuropeptide responses in layers remain unclear.

**Methods:**

Therefore, four trials were conducted to investigate the central effects of these amino acids on feed intake and hypothalamic neuropeptides in layer chicks. After intracerebroventricular administration, cumulative FI was measured over 2 hours and gene expression levels were determined using RT-PCR.

**Results:**

Our results showed that L-alanine (1 µg and 2 µg) decreased FI at 120 minutes post-injection and was associated with decreased neuropeptide Y (NPY) mRNA expression (*P* < 0.05). L-glutamine (3 µg) increased FI at 60 minutes post-injection (*P* < 0.05), but had no significant effect on proopiomelanocortin (POMC), NPY, melanocortin receptor 4 (MC4R) or agouti-related peptide (AgRP) (*P* > 0.05). L-arginine HCl (1 µg) decreased FI at 120 minutes post-injection, while increasing POMC and MC4R expression, and decreasing NPY (*P* < 0.05). Glycine (3 µg) significantly reduced FI at all measured time intervals (*P* < 0.05). It had no impact on POMC expression (*P* > 0.05), but increased MC4R expression (*P* < 0.05) and reduced NPY expression more than AgRP expression, although neither was significantly decreased (*P* > 0.05).

**Conclusion:**

These results suggest that the effect of individual amino acids on FI and hypothalamic neuropeptide expression in layer chicks depends on the type and dose of amino acid. The present findings provide evidence of the acute central effects of amino acids in layer chicks.

## 1 Introduction

Intracerebroventricular (ICV) injection of amino acids has been shown to affect feed intake regulation in chicks (Bungo and Shiraishi, 2010; Paul et al., 2012; Wang et al., 2012; Ahmadi et al., 2019; Kehinde et al., 2022; Ghashghaei et al., 2025b; Zarei et al., 2025a). Following central injection, compounds are delivered directly into the brain ventricles (McCosh and Young, 2024). Ventricles are located near appetite-regulating regions such as the hypothalamus, which has an important role in regulating signals related to appetite (Yousefvand and Hamidi, 2020; Yousefvand and Hamidi, 2022). Compounds may influence neuropeptides and their receptors in the hypothalamus. This approach is popular among researchers, and results related to physiological and behavioral responses can be obtained within 2–3 hours after injection. ICV experiments improve understanding of central responses and signaling pathways. They can also provide ideas for practical studies in poultry production.

Due to differences between strains, previous studies have been conducted using central injections in both broilers and layers (Griffin and Goddard, 1994; Contriciani et al., 2024). It has been shown that, although there are many similarities in central appetite regulation between broilers and layers, some differences exist, likely due to long-term selective breeding, indicating that each strain should be studied individually (Mahdavi et al., 2024).

A previous study reported that the hypothalamus in layers is sensitive to changes in environmental conditions, and under such conditions, the mRNA expression of appetite-related neuropeptides is altered, affecting feed intake in layers (Lozano-Villegas et al., 2025). It has been demonstrated that feed intake is regulated by amino acids, and this regulation varies between broilers and layers (Tran et al., 2019; Macelline et al., 2021). Therefore, it is important to determine how the brains of layers respond to compounds, including amino acids, and whether amino acids affect hypothalamic neuropeptides in layers to aid our understanding of their role in feed intake regulation in layers.

Administering L-alanine, L-glutamine, L-arginine and glycine ICV in avian models enables their direct role as neurochemical effectors within cerebral homeostatic centers to be elucidated, independently of their peripheral metabolic functions. Although these four amino acids have different primary degradation pathways, they are selected together because they converge to have an overall anorexigenic effect. Within the bird’s central nervous system (CNS), this combination of substrates interacts synergistically with the neuropeptidergic, monoaminergic and gaseous circuits of the hypothalamic complex to suppress food intake (Kurauchi et al., 2006).

A previous study showed that L-glutamine and L-alanine affect feed intake in broiler chickens by acting on hypothalamic neuropeptides, and that they are involved in the regulation of appetite (Paul et al., 2012). However, the main effects of these amino acids in laying hens, particularly in relation to hypothalamic neuropeptides, have not yet been investigated. Furthermore, central injection of arginine, an important amino acid involved in physiological processes, has been shown to lower feed intake in layers but not in broilers (Wang et al., 2012; Pourrahimi et al., 2018). However, it is unclear whether this reduction is related to changes in the expression of hypothalamic neuropeptides in layers. The impact of glycine, another amino acid involved in physiological functions, has been explored in both broilers and layers (Rahimi et al., 2021; Zarei et al., 2025a); however, its influence on hypothalamic neuropeptide expression remains unknown. These findings suggest that amino acids may influence feed intake depending on the type of amino acid and the chicken strain. However, information comparing their effects on hypothalamic neuropeptides in layers is limited. Therefore, information on how different amino acids affect hypothalamic neuropeptide responses in layer chicks is lacking. Closing this knowledge gap is critical because understanding the link between neuroendocrine regulation of appetite and feeding strategies could help to improve feed intake and increase productivity in layers (Wang et al., 2024).

Due to the differences between broilers and layers, we investigated the direct effects of L-alanine, L-glutamine, L-arginine monohydrochloride (L-arginine HCl) and glycine on feed intake and hypothalamic neuropeptides in layer chicks following an ICV injection. ICV administration was used to avoid gastrointestinal absorption. However, due to the bidirectional communication between the brain and peripheral organs, peripheral responses downstream of the central administration cannot be ruled out. Our hypothesis was that these amino acids affect feed intake by altering hypothalamic neuropeptide expression in layer chicks.

## 2 Materials and methods

### 2.1 Experimental animals

This study consisted of four trials, each examining the influence of an amino acid. Each experiment involved four groups (one control group and three groups that received different doses of amino acids), with eight chicks in each group. Consequently, 32 one-day-old male layer chicks (Hyland Brown) were used in each experiment to investigate the impact of amino acids. Furthermore, to minimize biological variation with respect to sex-related differences in the neonatal period, only male layer-type chicks were selected. While male and female chicks exhibit similar feeding-related physiological traits at this early stage of development, male layer chicks were selected for this experiment to maximize physiological and neurodevelopmental homogeneity, eliminate potential sex-dependent variations in the expression of central appetite-regulating neuropeptides, and maintain consistency with established avian ICV research protocols (Bungo, 1999). A total of 128 chicks were obtained from a local incubator (Shandong Tai’an Dongyue Poultry Production Co., Ltd.) to study the effect of the amino acids L-alanine, L-glutamine, L-arginine HCl and glycine on feed intake. The chicks were kept in a group cage for two days in a controlled environment with an optimum temperature (33 °C) and humidity (60%). At three days of age, each chick was moved to its own cage (Ghashghaei et al., 2025b). This individual housing (length: 42 cm, height: 35 cm, width: 11 cm) enabled us to accurately record the feed consumption of each chick, while preventing social interactions that could have affected differences in feed intake between individuals during the trial. Water and food (**Table 1**) were available from the first day until the fifth day, which was the day of injection (Ghashghaei et al., 2025a). Continuous lighting was provided for 24 h per day throughout the experimental period. The Animal Ethics Committee at Shandong Agricultural University authorized all stages of our work.

**Table 1:** Ingredient composition and nutrient content of the basal starter diet for layer-type chicks.

Item	Starter phase
Ingredients, %	
Corn (8.3% CP)	50.50
Soybean meal (43% CP)	33.00
Corn gluten meal (60% CP)	4.00
Wheat middling	5.00
Soybean oil	2.00
Salt	0.28
Limestone	1.75
Monodicalcium phosphate	1.55
Choline chloride	0.10
L-Lys·HCl (99%)	0.80
DL-Met (98%)	0.35
L-Thr (98%)	0.29
Probiotics + Enzyme	0.13
Vitamin premix^1^	0.05
Trace mineral premix^2^	0.20
Total	100.0
Nutrient composition	
Crude protein, %^4^	19.98
ME, kcal/kg^3^	2924
Total Ca, %^4^	0.88
Non-phytate P, %^3^	0.50
Lys, %^4^	1.25
Met, %	0.51
Met + Cys, %^3^	1.070
Thr, %^4^	0.68
Trp, %^3^	0.283

1 The vitamin premix provides the following quantities per kilogram of diet: vitamin A, 8000 IU; vitamin D3, 1000 IU; vitamin K, 0.5 mg; vitamin E, 20 IU; vitamin B12, 0.01 mg; cholecalciferol, 1300 IU; riboflavin 8 mg; niacin, 35 mg; pantothenic acid, 10 mg; biotin, 0.18 mg; folic acid, 0.55 mg.

2 The mineral premix provides the following quantities per kilogram of diet: Fe, 100 mg; selenium, 0.3 mg; copper, 8 mg; Zinc, 100 mg; I, 0.7 mg; manganese, 120 mg.

3 Nutrient composition calculated.

4 Nutrient composition measured.

### 2.2 Amino acids

The following amino acids and Evans Blue were purchased for this study: L-glutamine (C5H9NO4, MW: 146.14; Solarbio), L-alanine (C3H7NO2, MW: 89.09; Macklin), L-arginine HCl (C6H14N4O2, MW: 210.66; Sigma), glycine (C2H5NO2, MW: 75.07; Solarbio), and Evans Blue (C34H24N6Na4O14S4, MW: 960.81; SAIT). Evans Blue was dissolved in saline, and this solution was used as a control solution (0.9% saline containing 0.1% Evans Blue dye (w/v) to visually confirm the accuracy of the ICV injection) in the control group (Zendehdel et al., 2016). For the amino acid-injected groups, the amino acid was first dissolved in saline before being diluted with the control group solution (Zendehdel et al., 2017; Ghashghayi et al., 2022; Safikhani et al., 2023). We selected microgram-level doses for each amino acid based on earlier research in which ICV injections into layer-type chicks generated acute central responses (Alizadeh et al., 2015; Ghashghaei et al., 2025a). In addition, each chick received 10 microliters of amino acid solutions.

### 2.3 Procedures for ICV injection and feeding investigations

The experimental design was used to evaluate acute responses to ICV administration of amino acids in layer chicks. In each trial, when chicks were on the fifth day, those who were in the control group were injected with control solution (10 µL), and 3 other groups were injected with amino acid solution (1, 2, and 3 µg/10 µL) (**Table 2**). All solutions were adjusted to a pH of 7.4 ± 0.1 using 0.1 M NaOH or HCl. The calculated osmolarity of all treatment solutions remained strictly within the physiological isotonic range (SIM 308–312 mOsm/L), exerting no osmotic or pressure disruption on the central nervous system. The food bowl was taken from the cages at 8 a.m., and the food deprivation lasted three hours (Hesari et al., 2024; Rahmania et al., 2025), ensuring that all of the chicks were under identical conditions and had gone three hours without food. At 11 a.m., after three hours of food deprivation, the first chicken’s body weight was measured, and the first chicken was injected before receiving its feeding bowl. Similarly, after three hours of food deprivation, the body weight of other chicks was measured, chicks were injected, and food was given to them. The injection was carried out using an acrylic instrument. This device included a section where we could insert the chick’s head (Van Tienhoven and Juhász, 1962), and the head and beak were fastened. The solution was administered using a Hamilton syringe (Davis et al., 1979; Furuse et al., 1997) through a carefully positioned hole on a section of the device, such that when the syringe went through the hole and entered the chick’s brain, the solution would reach the third ventricle, which is adjacent to the hypothalamus. This method was used to provide the injections, which caused no stress to the chick (Furuse et al., 1997). While handling and restraint may cause a temporary stress response, the ICV procedure conducted in neonatal chicks is quick (overall less than 10 min) and has been widely used in previous studies. The time of restraint was kept to a minimum for stress-related effects. Furthermore, we refrained from anesthesia as anesthetic agents can affect central nervous system (CNS) activity and feed-responsive circuits potentially complicating the interpretation of acute feeding responses. Feed intake of each chick was measured at 30 minutes, 1 hour, and 2 hours after the injection. Two hours after injection, each chicken was beheaded and the hypothalamus tissue was collected. If blue dye was seen in the tissue, it suggested that the injected fluid had reached the hypothalamus (Badri et al., 2025; Zarei et al., 2025b). Thus, the tissue was removed and stored in liquid nitrogen. After collecting all hypothalamic tissues in liquid nitrogen, they were frozen at -80 °C until they were used for RNA extraction. All of our experimental chicks had blue dye in the hypothalamus, indicating that the injected fluid had reached the hypothalamic area. Cumulative feed intake was expressed relative to body weight to reduce the influence of body weight on the amount of food consumed by the chickens (Zarei et al., 2025c).

**Table 2:** Summary of dose equivalencies in nmol and mM.

Amino acid (MW)	Dose 1 µg	Dose 2 µg	Dose 3 µg
Glycine (75,07)	13.32 nmol/1.33 mM	26.64 nmol/2.66 mM	39.96 nmol/4.00 mM
L-Alanine (89,09)	11.22 nmol/1.12 mM	22.45 nmol/2.24 mM	33.67 nmol/3.37 mM
L-Glutamine (146,14)	6.84 nmol/0.68 mM	13.69 nmol/1.37 mM	20.53 nmol/2.05 mM
L-Arginine HCl (210,66)	4.75 nmol/0.47 mM	9.49 nmol/0.95 mM	14.24 nmol/1.42 mM

### 2.4 Extraction of RNA and quantification by RT-PCR

To investigate the effect of amino acids on hypothalamic neuropeptides, RNA was extracted from the hypothalamic tissue of chickens by Trizol (Invitrogen) in a completely sterile environment, and its concentration was measured by a spectrophotometer. Reverse transcription was performed with a cDNA synthesis kit (Cwbio, China). RT-PCR was performed with the Magic SYBR Mixture (Cwbio, China). The primers (**Table 3**) used in this study were previously designed by the NCBI Primer BLAST program. Normalization of gene expression was performed with the internal gene GAPDH (Ghashghaei et al., 2025b), and its amount was determined by the comparative CT method (2 − ΔΔCT) (Ghashghaei et al., 2025c).

**Table 3:** Primers for the targeted and reference transcripts (Ghashghaei et al., 2025a).

Gene	Primer sequence
NPY	F: GAGGCACTACATCAACCTCATCAC R: TGTTTTCTGTGCTTTCCCTCAA
MC4R	F: ACACTCCAGCCTCTCCATTTCT R: TGTTCATAGCAGCCTCCCGA
AgRP	F: GGAACCGCAGGCATTGTC R: GTAGCAGAAGGCGTTGAAGAA
POMC	F: CGCTACGGCGGCTTCA R: TCTTGTAGGCGCTTTTGACGAT
GAPDH	F:ACATGGCATCCAAGGAGTGAG R:GGGGAGACAGAAGGGAACAGA

### 2.5 Statistical analysis

The effects of amino acids on cumulative feed intake were analyzed using a two-way ANOVA with GraphPad Prism software (version 8.0.2). Feed intake was repeatedly measured for each chick at 30, 60 and 120 minutes post-injection; therefore, time was considered a repeated factor in the analysis. The primary factors were treatment (amino acid dose) and time, and their interactions were examined. The study included data from the control group, as well as from groups that received three doses of amino acids at three different times. Each group consisted of eight chicks, with each bird constituting a replicate. Thus, eight data points were collected for each group. The Tukey-Kramer test was used to perform multiple comparisons, with statistical significance set at *P* < 0.05. In the gene expression study, Student’s t-tests were used to evaluate independent samples, with a statistical significance threshold set at *P* < 0.05. As gene expression was measured at only one time point, each amino acid treatment was only compared to its own control group (e.g. the control for L-alanine was compared to its treatment dose, and the control for L-arginine hydrochloride was compared to its treatment dose). Therefore, comparisons were made between each treatment group and its corresponding control.

## 3 Results

### 3.1 Central effects of amino acids on feed intake of laying chicks

Experiments 1–4 investigated the impact of individual amino acids on feed intake in laying chicks, examining the effects of 1 µg, 2 µg and 3 µg doses administered over a two-hour period post-injection. Experiment 1 showed that L-alanine decreased feed intake at all doses, but this reduction was only significant at the 1 µg and 2 µg doses after 2 hours (*P* < 0.05; **Figure 1**). Experiment 2 found that L-glutamine at a dose of 3 µg increased feed intake at all time points, although the effect was only significant after 60 minutes (*P* < 0.05; **Figure 2**). Experiment 3 found that L-arginine HCl at a dose of 1 µg reduced feed intake at three time points, though this was only significant at two hours post-injection (*P* < 0.05; **Figure 3**). Experiment 4 found that glycine at a dosage of 3 µg significantly reduced feed intake at all measured time intervals (*P* < 0.05; **Figure 4**). All measurements were performed up to two hours post-injection, reflecting the acute central effects of each amino acid.

**Figure 1:**
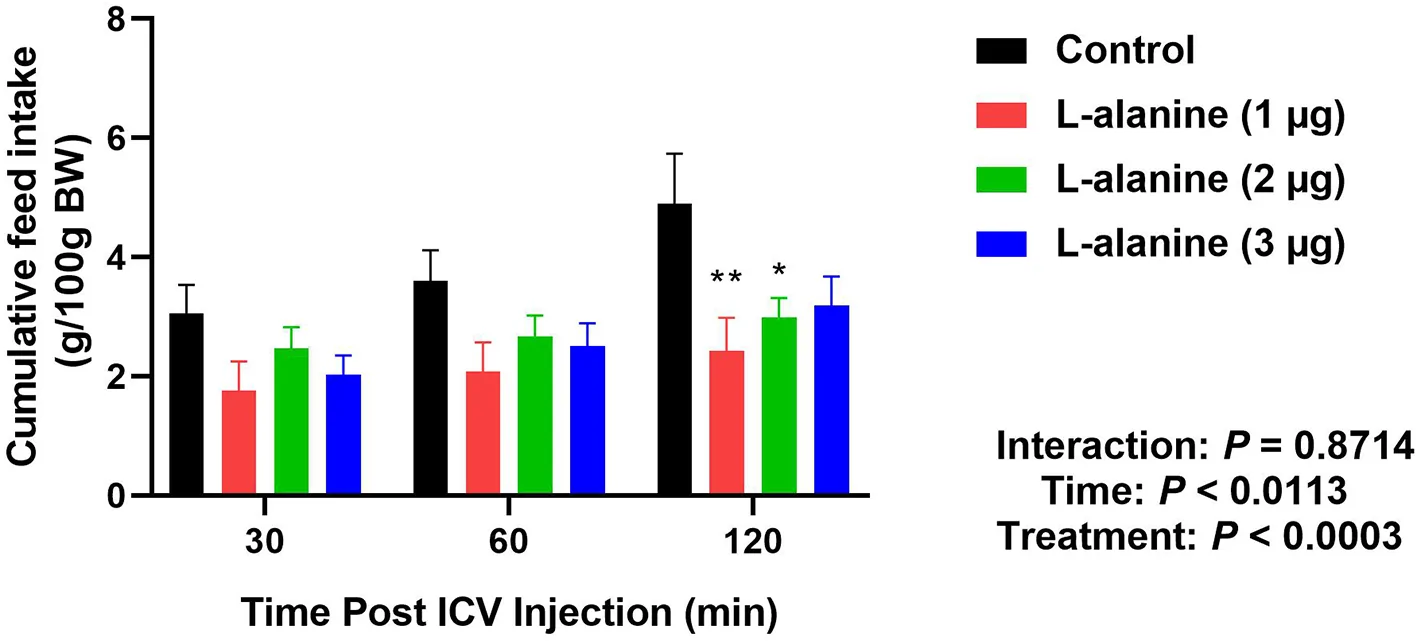
The impact of centrally administered L-alanine at doses of 1, 2, and 3 μg on the feed intake of layer-type chicks. Measurements of feed intake were taken at 30, 60, and 120 minutes following central injection. Values are reported as mean ± SEM (n = 8). *Significant differences are indicated by *P < 0.05 and **P < 0.05.

**Figure 2:**
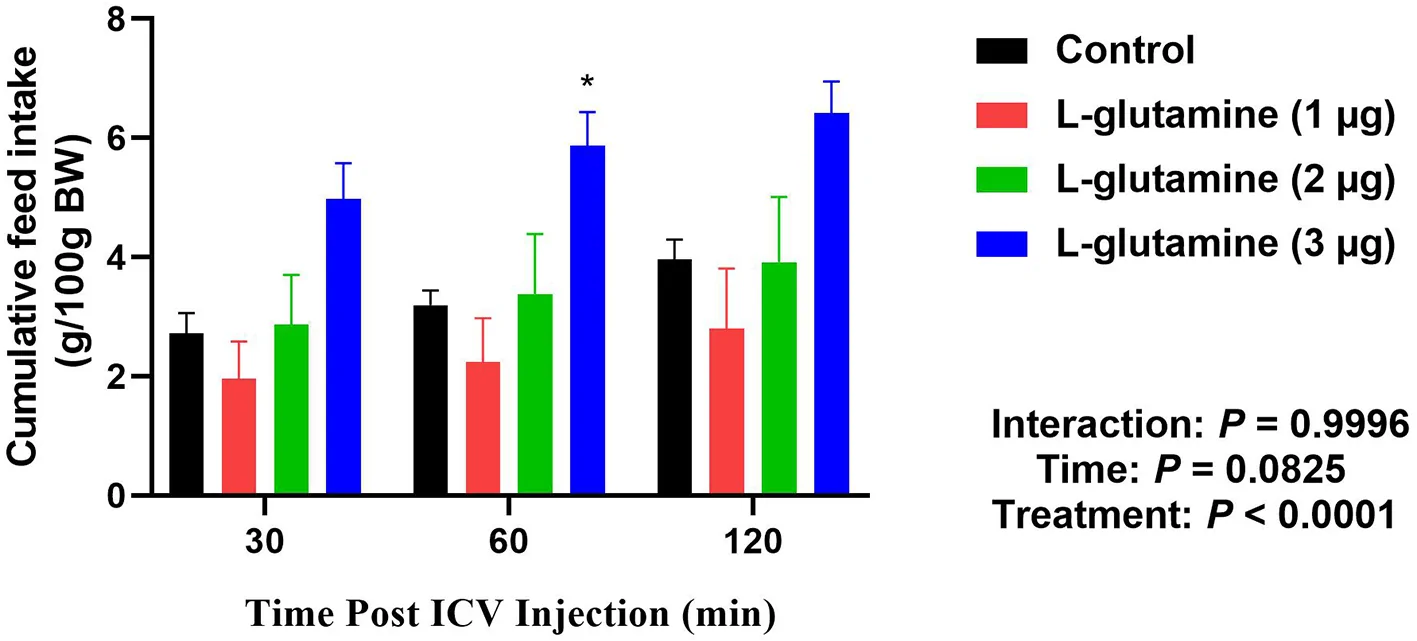
Effects of centrally administered L-glutamine (1, 2, and 3 μg) on feed intake in layer-type chicks. Measurements of feed intake were taken at 30, 60, and 120 minutes after injection. Values are reported as mean ± SEM (n = 8), with a significance level of *P < 0.05.

**Figure 3:**
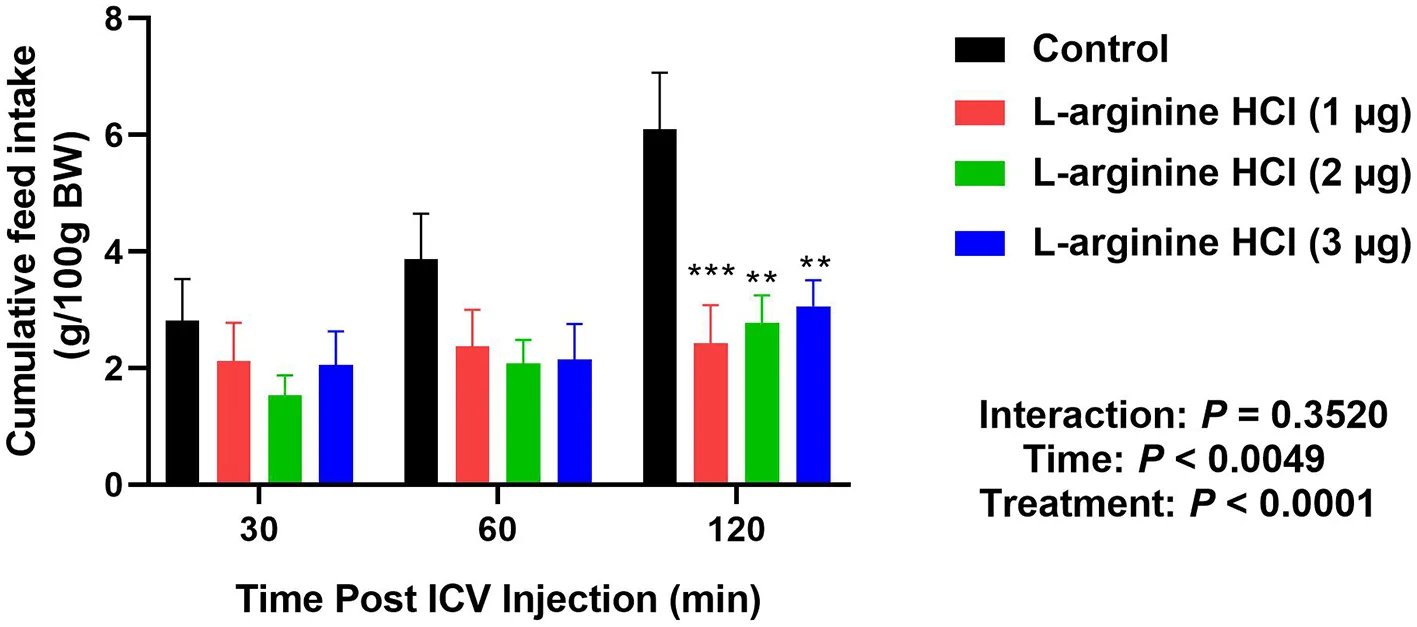
Effects of centrally administered L-arginine HCl (1, 2, and 3 μg) on feed intake in layer-type chicks. Measurements of feed intake were taken at 30, 60, and 120 minutes after injection. Values are reported as mean ± SEM (n = 8). *Significant differences are indicated by **P < 0.05 and ***P < 0.05.

**Figure 4:**
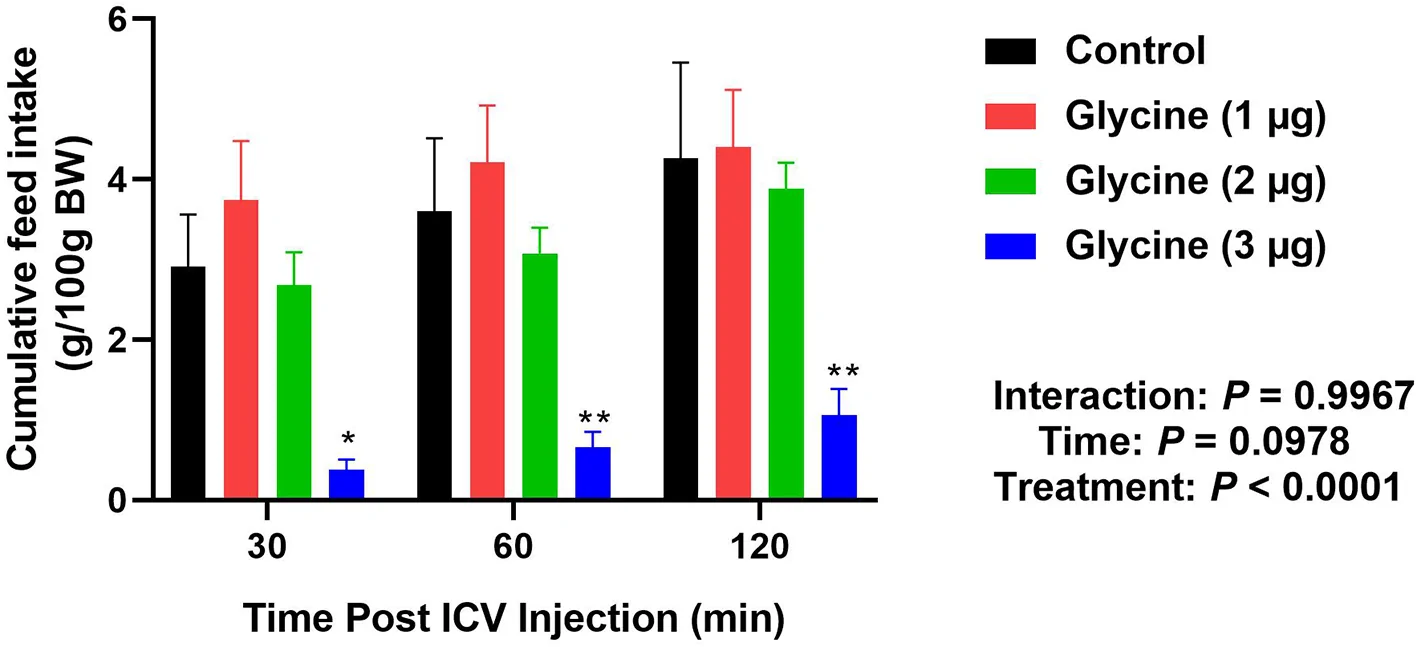
Effects of centrally administered glycine (1, 2, and 3 μg) on feed intake in layer-type chicks. Measurements were conducted at 30, 60, and 120 minutes following central injection. Values are reported as mean ± SEM (n = 8). *Significant differences are indicated by **P < 0.05 and *P < 0.05.

### 3.2 Central effects of amino acids on mRNA expression of appetite-related neuropeptides

Gene expression analysis was performed using the doses that elicited the greatest feed intake responses. Each amino acid was only compared to its own control. As gene expression was measured at a single time point, comparisons were made between each treatment group and its respective control group.

L-alanine: Gene expression was analyzed for the 1 µg dose, as the group injected with this dose showed lower feed intake than the 2 µg group. Pro-opiomelanocortin (POMC) and melanocortin receptor 4 (MC4R) mRNA levels increased slightly, but this was not significant (*P* > 0.05). Neuropeptide Y (NPY) mRNA expression decreased significantly (*P* < 0.05; **Figure 5**). Agouti-related peptide (AgRP) mRNA expression remained unchanged (*P* > 0.05).

**Figure 5:**
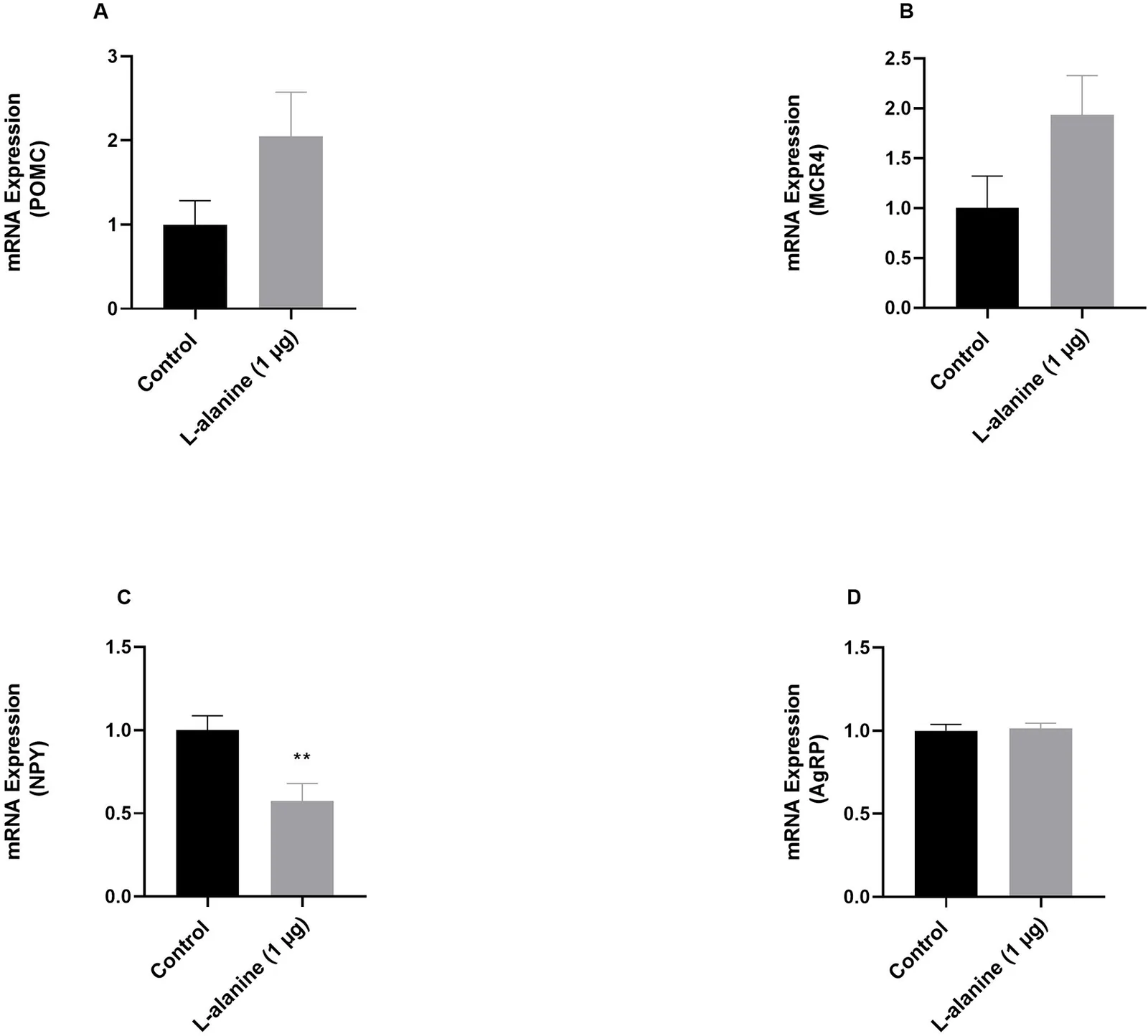
The impact of centrally delivered L-alanine at doses of 1 μg on the expression of genes involved in appetite was examined in chicks. Values are reported as mean ± SEM (n = 8), with a significance level of *P < 0.05. **(A)** POMC (pro-opiomelanocortin), **(B)** MC4R (melanocortin receptor 4), **(C)** NPY (neuropeptide Y), and **(D)** AgRP (agouti-related peptide).

L-glutamine: At a dose of 3 µg, L-glutamine was found to slightly decrease anorexigenic gene expression (POMC and MC4R) and slightly increase appetitive gene expression (NPY and AgRP). However, none of these changes were statistically significant (*P* > 0.05; **Figure 6**).

**Figure 6:**
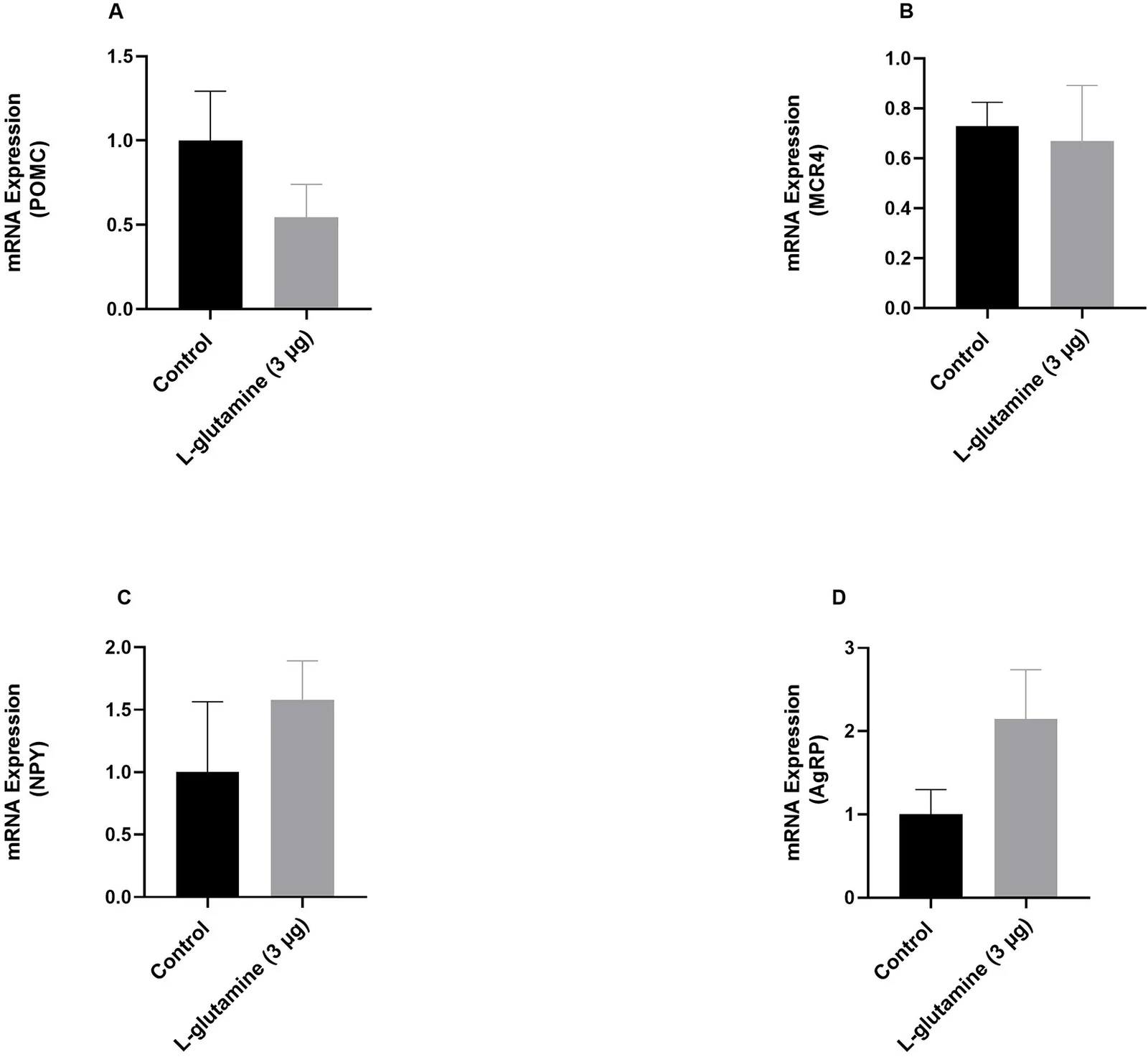
The impact of centrally delivered L-glutamine (3 μg) on the expression of hypothalamic genes involved in appetite regulation was examined in neonatal layer-type chicks. The data are reported as mean ± SEM (n = 8). No comparisons reached statistical significance. **(A)** POMC (pro-opiomelanocortin), **(B)** MC4R (melanocortin receptor 4), **(C)** NPY (neuropeptide Y), and **(D)** AgRP (agouti-related peptide).

L-arginine HCl: At a dose of 1 µg, L-arginine HCl reduced feed intake. Gene analysis showed upregulation of POMC and MC4R, as well as downregulation of NPY (*P* < 0.05; **Figure 7**). AgRP decreased slightly, but this change was not significant (*P* > 0.05; **Figure 7**).

**Figure 7:**
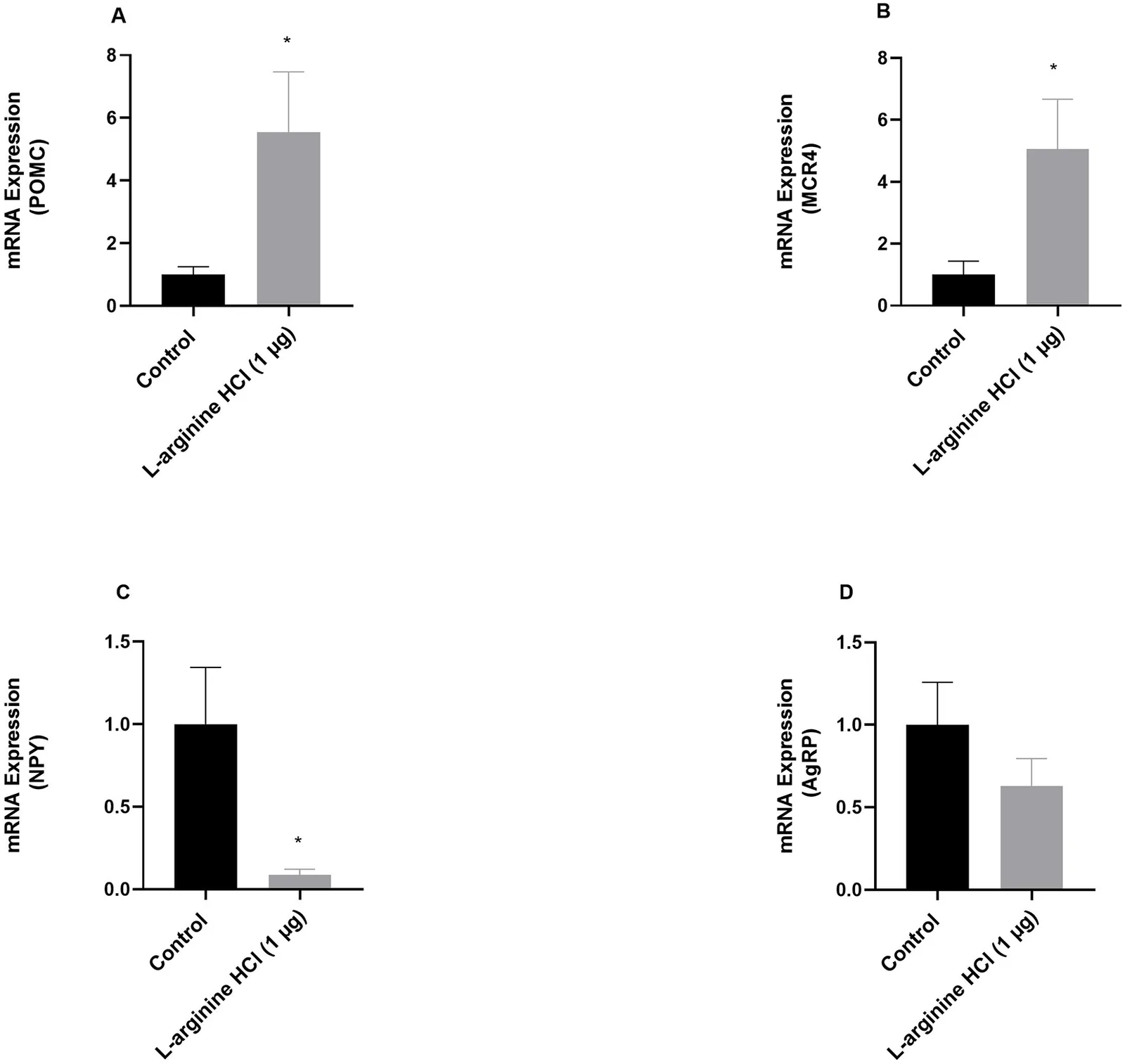
The impact of centrally delivered L-arginine HCl (1 μg) on the expression of hypothalamic genes involved in appetite regulation was examined in neonatal layer-type chicks. The data are presented as mean ± SEM (n = 8). *Significant differences are indicated by *P < 0.05. **(A)** POMC (pro-opiomelanocortin), **(B)** MC4R (melanocortin receptor 4), **(C)** NPY (neuropeptide Y), and **(D)** AgRP (agouti-related peptide).

Glycine: At 3 µg, glycine had no effect on POMC, significantly increased MC4R (*P* < 0.05; **Figure 8**) and reduced NPY by a greater amount than AgRP; however, neither of these decreases was significant (*P* > 0.05; **Figure 8**).

**Figure 8:**
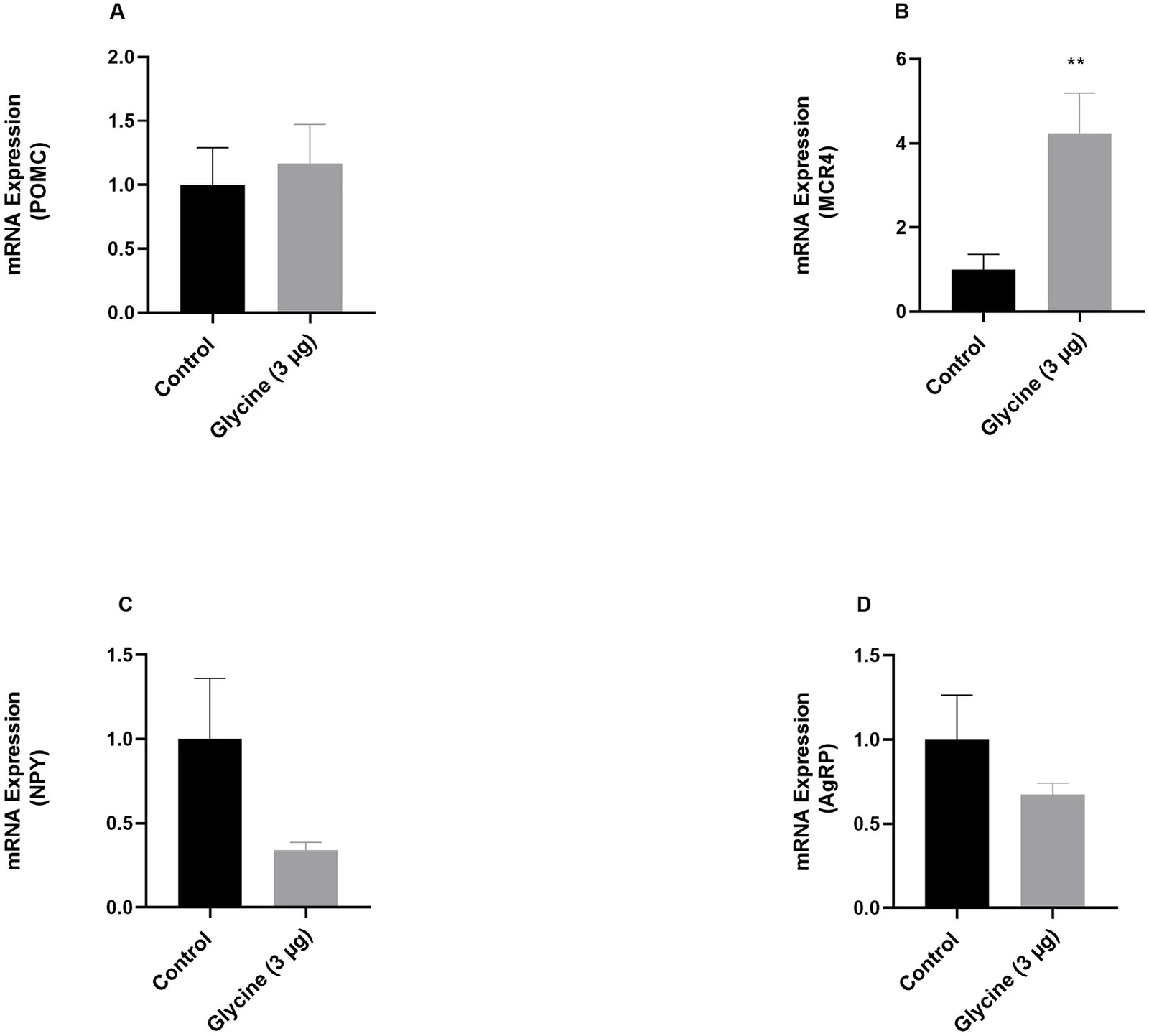
The impact of centrally delivered glycine (3 μg) on the expression of hypothalamic genes involved in appetite regulation was examined in neonatal layer-type chicks. The data are presented as mean ± SEM (n = 8), with a significance level of **P < 0.05. **(A)** POMC (pro-opiomelanocortin), **(B)** MC4R (melanocortin receptor 4), **(C)** NPY (neuropeptide Y), and **(D)** AgRP (agouti-related peptide).

All gene expression measurements reflect acute effects and were evaluated relative to the corresponding control group for each amino acid.

## 4 Discussion

Analysis of hypothalamic gene expression reveals that, despite an overall hypophagic effect being shared by all three amino acids, L-alanine, L-arginine HCl and glycine employ distinct and specific neurochemical signatures to modulate feed intake in 5-day-old chicks. These results confirm our hypothesis that amino acids influence feed intake by altering the expression of hypothalamic neuropeptides in laying chicks. This study provides new insights into the central regulation of appetite in laying chicks, whose metabolic requirements and neuroendocrine regulation of energy balance differ from those of broiler chickens (Druyan, 2010; Buzała et al., 2015; Contriciani et al., 2024; Ghanem et al., 2024). However, only gene expression was measured, so the underlying mechanisms remain unclear. Therefore, to understand the central mechanisms of appetite in laying chicks, studies focusing on these chicks are necessary rather than relying on results obtained in broiler chickens. This study addresses the lack of information regarding the central effects of these four amino acids in laying chicks, which have distinct metabolic needs and a different neuroendocrine regulation of energy balance.

According to our results, L-alanine directly attenuated central hunger drive by downregulating orexigenic NPY mRNA expression (P < 0.05); glycine increased satiety sensitivity by upregulating MC4R expression (P < 0.05); and L-arginine elicited a dual response by significantly repressing NPY and activating the anorexigenic melanocortin axis (POMC and MC4R upregulation, P < 0.05). In contrast, central administration of L-glutamine had an orexigenic effect, significantly stimulating feed intake (P < 0.05). As transcriptional shifts in NPY, AgRP, POMC and MC4R did not reach statistical significance (P > 0.05), the acute feeding stimulation caused by L-glutamine is likely to be mediated by rapid synaptic neurotransmission, acting as a direct metabolic precursor for central glutamate and GABA pool dynamics rather than by immediate gene transcription. In birds, NPY is one of the most potent orexigenic peptides. The ability of L-alanine to specifically repress the NPY pathway suggests that its acute hypophagic effect does not depend on the activation of the melanocortin satiety system, but rather on the suppression of the hunger signal. This finding supports the hypothesis that L-alanine acts as a cellular energy sensor at the hypothalamic level, whereby central delivery of this amino acid could mimic a state of nutritional abundance and selectively signal to NPY neurons to curb food-seeking behavior (Ghashghaei et al., 2025b). Furthermore, the effect of L-alanine on both broilers and layers was to reduce feed intake. However, a previous study showed that the reduction in broilers was associated with a decrease in AgRP and an increase in MC4R expression (Paul et al., 2012), whereas in our study of layers, the reduction was associated only with a decrease in NPY. These differences suggest that the mechanisms underlying the effect of L-alanine on feed intake may vary between broilers and layers.

Administering 3 µg of L-glutamine 60 minutes after injection significantly affected food intake, but had no significant effect on the expression of the POMC, NPY, MC4R or AgRP genes. In fact, there were slight inverse trends (**Figure 6**). These results suggest that L-glutamine may influence food intake via mechanisms other than transcriptional regulation, which were not fully investigated in this study. One possible explanation is that L-glutamine is a precursor of glutamate. After reaching the hypothalamus following ICV injection, it could be converted to glutamate. Glutamate is known to act as a rapid orexigenic mediator in the hypothalamus (Stanley et al., 1993; Wang et al., 2012; Ahmadi et al., 2019). Furthermore, a previous study reported that a compound could influence food intake without transcriptional regulation (Proszkowiec-Weglarz et al., 2017). When the effects of L-glutamine were compared in the two strains (broiler chickens and laying chicks), no significant changes in neuropeptide expression were observed in laying hens in our study. However, broiler chickens showed significant increases in NPY and decreases in POMC (Paul et al., 2012), inducing a similar stimulatory effect on feed intake. These results further suggest that different hypothalamic pathways can regulate similar behavioral responses in different strains.

Compared to L-alanine and L-glutamine, L-arginine hydrochloride (1 µg) had the most significant impact on hypothalamic gene expression. It increased the expression of the POMC and MC4R genes and significantly decreased the expression of the NPY gene (**Figure 7**). This suggests strong activation of satiety-related signals and suppression of appetite-related signals. POMC, MC4R and NPY have been shown to play an important role in regulating food intake in birds (Bungo et al., 2011; Scanes and Pierzchała-Koziec, 2021; Greene et al., 2022). In chicks, L-arginine is the sole substrate of neuronal nitric oxide synthase (nNOS). While our study did not measure this parameter, we hypothesize that there was strong local production of nitric oxide (NO) induced by the intracerebroventricular injection of L-arginine. This NO could have acted as a potent retrograde mediator capable of simultaneously activating POMC/MC4R anorexigenic neurons and inhibiting the NPY orexigenic network within the avian hypothalamus. However, previous studies have reported decreased feed intake in laying hens (Pourrahimi et al., 2018) and no effect of L-arginine on feed intake in broiler chickens (Wang et al., 2012). Our results suggest that the behavioral effect of L-arginine hydrochloride on feed intake in laying hens may be related to changes in hypothalamic hunger and satiety signals. It should be noted that the dose-response relationship was limited, as only a few concentrations were tested.

Finally, at a dose of 3 µg, glycine exhibits a unique mechanism of action. Its effect on the hypothalamus differs from that of L-arginine hydrochloride, suggesting that its impact on feed intake is primarily due to MC4R receptor activation rather than changes in neuropeptide levels. Selective MC4R upregulation indicates that glycine directly sensitizes or activates signaling pathways downstream of the melanocortin system that influence feed intake (Garfield et al., 2015). This is a plausible mechanism for the effect of glycine observed in our study. In poultry, central glycine interacts closely with N-methyl-D-aspartate (NMDA) receptors and the adrenergic system. The increase in MC4R could reflect synaptic convergence, whereby glycine potentiates satiety circuits independently of POMC transcription. This illustrates the rapid transcriptional plasticity of hypothalamic receptors in response to an influx of inhibitory amino acids. Our results concerning the effect of glycine on feed intake are consistent with previous research demonstrating that glycine can reduce feed intake in both broiler chickens (Rahimi et al., 2021) and laying hens (Zarei et al., 2025a). Together, these findings suggest that glycine may reduce feed intake by targeting receptors that regulate appetite, rather than by altering neuropeptide expression.

Gene expression analysis was performed for each amino acid relative to the corresponding control group. All reported effects are acute, as measurements were taken up to two hours post-injection and long-term effects were not evaluated in this study. It should also be noted that, due to the difference in molecular mass between glycine (75.07 g/mol), L-alanine (89.09 g/mol), L-glutamine (146.14 g/mol) and L-arginine hydrochloride (210.66 g/mol), administering doses of identical mass results in variations in the absolute molar quantities introduced into the ventricular system (**Table 2**). For instance, at the maximum dose of 3 µg, the administered molar load differs significantly: 39.96 nmol for glycine, 33.67 nmol for L-alanine, 20.53 nmol for L-glutamine and just 14.24 nmol for L-arginine HCl (a ratio of almost 3:1 between glycine and L-arginine). This disparity in molar quantities has important implications for interpreting the relative potency of each amino acid. The significant anorexigenic effect observed with higher molecular weight amino acids (such as L-arginine and L-glutamine), despite a molar load that is approximately three times lower, strongly suggests greater sensitivity or receptor affinity in avian neuronal networks (such as the nitrergic and melanocortin systems) for these specific molecular structures. Conversely, the effect of glycine could be underpinned by its higher molar density, which activates ionotropic receptors (GlyRs) and NMDA receptors more broadly. Therefore, our results should be interpreted as an assessment of the overall behavioral efficacy induced by mass loading of nutrients, rather than as a comparison of intrinsic stoichiometric affinity for a single receptor.

In conclusion, our results suggest that the observed overall hypophagic effect is not the result of a single common pathway, but rather the outcome of various molecular strategies. L-alanine inhibits hunger (neuropeptide Y, NPY), glycine stimulates satiety sensitivity (melanocortin 4 receptor, MC4R), and L-arginine orchestrates an integrated response on both fronts. In contrast, central L-glutamine administration significantly stimulated feed intake, representing an orexigenic signal. By demonstrating that individual central amino acids act as neurochemical switches that modulate key hypothalamic appetite genes (NPY, POMC and MC4R), these results could inform the development of nutritional strategies in poultry production. In practice, identifying these neuroendocrine targets provides proof of concept for formulating functional early-life feeds, such as pre-starter diets or in-ovo supplements. For example, L-glutamine could be utilized to stimulate early post-hatch feed intake, while L-arginine and glycine could be used to manage metabolic intake under stressful conditions. To translate these central mechanisms into commercial production systems, future studies should evaluate the oral dietary delivery of these amino acids and their long-term effects on growth performance.

## Data Availability

The original contributions presented in the study are included in the article/supplementary material. Further inquiries can be directed to the corresponding author.
